# (Poly)phenols in Inflammatory Bowel Disease and Irritable Bowel Syndrome: A Review

**DOI:** 10.3390/molecules26071843

**Published:** 2021-03-25

**Authors:** Marilyn Hagan, Bu' Hussain Hayee, Ana Rodriguez-Mateos

**Affiliations:** 1 Department of Nutrition and Dietetics, Royal Papworth Hospital NHS Foundation Trust, Cambridge CB2 0AY, UK; marilyn.hagan@nhs.net; 2 Department of Gastroenterology, King’s College Hospital NHS Foundation Trust, London SE5 9RS, UK; b.hayee@nhs.net; 3 Department of Nutritional Sciences, Faculty of Life Sciences and Medicine, King’s College London, London WC2R 2LS, UK

**Keywords:** inflammatory bowels disease (IBD), Crohn’s disease, ulcerative colitis, irritable bowel syndrome (IBS), (poly)phenols

## Abstract

(Poly)phenols (PPs) may have a therapeutic benefit in gastrointestinal (GI) disorders, such as irritable bowel syndrome (IBS) or inflammatory bowel disease (IBD). The aim of this review is to summarise the evidence-base in this regard. Observational evidence does not give a clear indication that PP intake has a preventative role for IBD or IBS, while interventional studies suggest these compounds may confer symptomatic and health-related quality of life improvements in known patients. There are inconsistent results for effects on markers of inflammation, but there are promising reports of endoscopic improvement. Work on the effects of PPs on intestinal permeability and oxidative stress is limited and therefore conclusions cannot be formed. Future work on the use of PPs in IBD and IBS will strengthen the understanding of clinical and mechanistic effects.

## 1. Introduction

(Poly)phenols (PPs, including phenolic compounds with at least one aromatic ring with one or more hydroxyl groups attached), are a very large group of plant secondary metabolites that are sub-classified as flavonoids and non-flavonoids. Flavonoids comprise flavan-3-ols, flavones, isoflavones, flavonols, dihydroflavonols, flavanones, proanthocyanins, anthocyanins, chalcones, dihydrochalcones, and aurones. Non-flavonoids include phenolic acids (hydroxybenzoic acids and hydroxycinnamic acids), stilbenes, lignans, coumarins, curcuminoids, and tannins (such as ellagitannins) [1,2,3]. The most important dietary sources include coffee, tea, fruits and vegetables, cocoa, soy products, nuts, olive oil, red wine, cereals, and wholegrains [2,3,4]. PPs have been ascribed anti-inflammatory and cardio-protective effects (amongst others) [1,2,5] and it has therefore been proposed that they may be beneficial in gastrointestinal (GI) disorders such as inflammatory bowel disease (IBD) and irritable bowel syndrome (IBS) [6,7].

IBD comprises Crohn’s disease (CD) and ulcerative colitis (UC), which are both chronic inflammatory conditions of the GI tract [8]. Globally, there are 6.8 million people living with IBD (84.3 per 100,000 population), with the highest reported prevalence in North America followed by the United Kingdom [9]. The chronic and intermittent nature of IBD means daily physical, social, and professional activities can be severely impacted [10]. Abdominal pain, abdominal cramping during bowel movements, and blood, pus, or mucus in stool are common symptoms [11]. Pharmacological treatments for the induction and maintenance of remission in IBD include aminosalicylates, corticosteroids, immuno-modulators, and monoclonal antibodies [11]. Pharmacological treatments have a variety of adverse effects including gastrointestinal disturbance, headaches, ache, weight loss, increased risk of infections, diabetes, and bone mass loss, amongst others [12,13]. The literature has promoted several diets to induce remission in IBD, such as the specific carbohydrate diet (SCD), low fermentable oligosaccharide, disaccharide, monosaccharide, and polyol (FODMAP) diet, the Palaeolithic diet (Paleo), and the anti-inflammatory diet (IBD-AID) [14]. The SCD diet recommends consumptions of monosaccharides and restriction of disaccharides and polysaccharides based on the theory that disaccharides and polysaccharides pass into the colon undigested, causing intestinal injury [14]. The Paleo diet suggests exposure to foods not present during human evolution may result in modern diseases; therefore, a lean protein and high fibre plant-based diet is recommended [15]. Excessive intake of FODMAPs, which are highly fermentable but poorly absorbed short-chain carbohydrates and polyols, can increase intestinal permeability [16] as well, producing significant symptomatic gastrointestinal upset. The low FODMAP diet involves eliminating foods high in FODMAPs for 6–8 weeks and gradually reintroducing them with the support of a dietitian [17]. Unblinded and observational studies suggest that reducing FODMAPs in IBD patients in remission could convey benefits for IBS-like symptoms [18]. Randomised control trial data is lacking; therefore, professional consensus does not currently recommend “IBD diets” to promote remission in patients with active disease [19].

IBS is a functional gastrointestinal disorder defined using the Rome IV criteria as a combination of abdominal pain, disordered bowel habits, and bloating [20]. Onset of symptoms should occur at least 6 months prior to diagnosis and have been present for the preceding 3 months [20]. IBS can be classified into four subtypes according to the most predominant symptom: constipation-predominant (IBS-C), diarrhoea-predominant (IBS-D), mixed bowel habits (IBS-M), or unsubtyped [21]. The prevalence of IBS is estimated at 5.7% in the United States, Canada, and the United Kingdom [22]. Several factors have been implicated in the pathogenesis of IBS, including visceral hypersensitivity, brain-gut interactions, post infectious reactivity, intestinal inflammation, food sensitivity, carbohydrate malabsorption, altered gastrointestinal motility, bacterial overgrowth, and alterations in faecal microflora [23]. Changes in bacterial microflora, food allergy, infectious enteritis, and genetic factors may contribute to low grade inflammation, but the mechanisms are not fully understood [24]. Treatment of IBS predominantly involves dietary and lifestyle modification [25,26]; however, medication can be used to treat individual symptoms of cramping, constipation, and diarrhoea [27]. Diet and lifestyle consensus guidance is based on very low to moderate evidence from randomised controlled trials (RCTs) and controlled trials and includes regular meal patterns, modification of dietary fibre and fluids, and low FODMAP diet [26]. Pharmacological consensus guidance is also based on very low to moderate evidence from RCTs and includes antispasmodic agents, laxatives for constipation, and antimotility agents for diarrhoea. Tricyclic antidepressants (TCAs) are considered second line treatment followed by selective serotonin reuptake inhibitors (SSRI) [26].

There is a growing body of in vitro and animal model evidence on benefits of PPs in controlling cytokine-mediated inflammation, immune signalling, and free radical activity that are implicated in IBD and IBS pathogenesis [7,28]. More recently, some evidence from human studies on the efficacy of PPs in IBD and IBS has been published, including meta-analyses on the use of curcumin in UC and CD, which is the type of (poly)phenol most commonly investigated so far [29,30,31]. Previous reviews on this topic have focused on individual PPs, mechanisms of action, and evidence from preclinical studies [7,32,33,34,35]. The aim of this article is to review the existing and emerging evidence from human studies for the use of PPs on the prevention and management of IBD and IBS, with a focus on patient outcomes.

## 2. (Poly)phenols, Inflammatory Bowel Disease, and Irritable Bowel Syndrome

A total of 27 interventional studies investigating the effect of PPs in IBD and IBS are included in this review (Table 1, Table 2 and Table 3). Of the 18 IBD studies, 15 were randomized controlled trials (RCTs), while 3 were uncontrolled trials, ranging from 1 week to 6 months of duration, with 18–83 individuals enrolled. Regarding intervention studies in IBS, 8 were RTCs and 1 was a supplement registry ranging from 4–12 weeks of duration, and included 44 to 241 subjects. A total of 11 studies investigated the efficacy of curcumin, while other PP sources studied included pure compounds (resveratrol, epigallocatechin-3-gallate (EGCG), isoflavones, proanthocyanidins, and silymarin) and PP-rich foods (red wine, mango, flaxseeds, ginger, green tea, bilberry, olive oil, and green tea).

Only 10 small observational studies were included and summarized in Table 4 (6 cross-sectional and 4 case-control studies), with 6 IBD studies including 55–256 individuals and 4 IBS studies with 297–388 subjects. The studies estimated the dietary intake of isoflavones, proanthocyanidins, total polyphenols, tea, coffee, or chocolate using food or lifestyle factor questionnaires.

A variety of outcome measures were tested in the studies including IBD and IBS related symptoms, inflammatory markers, health-related quality of life, incidence of IBS and IBD, intestinal permeability, endoscopic response, and oxidative stress. The main findings and conclusions of these studies are discussed in the Section 2.1, Section 2.2, Section 2.3, Section 2.4, Section 2.5, Section 2.6 and Section 2.7.

### 2.1. (Poly)phenols and Symptoms in Inflammatory Bowel Disease and Irritable Bowel Syndrome

A total of 21 studies investigated the effects of PPs on IBD symptoms (Table 1, Table 2 and Table 4), including curcumin (*n* = 9), bilberry anthocyanin (*n* = 1), green tea EGCG (*n* = 1), extra virgin olive oil (*n* = 1), flaxseed (*n* = 1), ginger (*n* = 1), silymarin (*n* = 1), pure trans-resveratrol (*n* = 1), mango (*n* = 1), red wine (*n* = 1), proanthocyanidins (*n* = 1), and isoflavones (*n* = 2).

The Mayo score, ulcerative colitis disease activity index (UCDAI), and disease activity index (DAI) are four-criteria disease activity indices in UC that include stool frequency, rectal bleeding, appearance of mucosa on sigmoidoscopy, and a physician’s assessment of disease severity [75,76]. The partial Mayo score includes the 3 criteria of the Mayo score excluding the endoscopic component [77]. Significant reduction in UCDAI and clinical remission defined as UCDAI ≤ 3 was correlated with the administration of green tea EGCG [45] and curcumin enema [42]. However, a low dose of oral curcumin administration (450 mg for 8 weeks) did not establish an improvement in UCDAI [41]. Silymarin contains flavanolignans and significantly improved DAI at a dose of 240 mg/day for 6 months [49]. Bilberry anthocyanins (840 mg/day for 6 weeks) [44], flaxseed, and flaxseed oil (30 g/day and 10 g/day respectively for 12 weeks) [46] significantly reduced the Mayo score; however, extra virgin olive oil (50 mL/day for 20 days) had no effect [47]. Curcumin supplementation (3000 mg/day for 1 month) significantly reduced the partial Mayo score by ≥1 point [38]. As the indexes mentioned use similar rating scales, they collectively support the use of higher doses of oral curcumin, curcumin enema, green tea EGCG, silymarin, bilberry anthocyanin, flaxseed, and flaxseed oil for reducing symptoms in UC.

The simple clinical colitis activity index (SSCAI) is a five-criteria disease activity index including bowel frequency in the day and night, urgency of defecation, blood in stool, general wellbeing, and extra-colonic features [78]. In UC, a significant reduction in SSCAI was demonstrated with the administration of 3000 mg curcumin for 1 month [38], 240 mg curcuminoids nanomicelles for 28 days [39], pure trans-resveratrol [50,51], mango [52], and ginger powder [48]. Clinical remission defined as a SCCAI ≤ 2 was significantly correlated with curcumin supplementation of 1500 mg-3000 mg/day in UC [38,40]. Mango administration also significantly reduced SSCAI in CD [52]. The clinical activity index (CAI) is a 7 criteria disease activity index including number of stools per week, blood in stools, abdominal pain/cramps, temperature, lab findings (erythrocyte sedimentation rate (ESR) and haemoglobin concentration), and general wellbeing [79]. In UC, CAI significantly reduced with bilberry anthocyanin supplementation [44] and both curcumin alone [37] and curcumin administered in combination with green tea [36]. The gastrointestinal symptom rating scale (GSRS) is a 15 item rating scale used to assess symptoms in IBD and peptic ulcer disease [80]. Consumption of extra virgin olive oil significantly reduced the GSRS in participants with UC [47]. One study investigating curcumin supplementation found a significant reduction in global score that considered general wellbeing, number and quality of stool, blood in stool, abdominal pain, rectal pain urgency, medication change, and endoscopy [43].

The Crohn’s disease activity index (CDAI) is a seven-criteria disease activity index including frequency of liquid stools, abdominal pain, general wellbeing, presence of complication, use of medication for diarrhoea, abdominal mass, haematocrit, and deviation from standard weight [81]. Administration of Theracurmin, which has increased bioavailability in comparison with standard curcumin, resulted in a significant reduction in CDAI [54] compared with a non-significant reduction with standard curcumin [43]. One study found no change in CDAI in CD or ulcerative colitis clinical activity index (UCAI) in UC with moderate red wine consumption [53]. This study paper referred to UCAI but did not specify which standard index was used, therefore it is difficult to compare results with other studies investigating symptoms in UC.

The impact of PPs on individual gastrointestinal symptoms in UC is also explored in the literature, although data is scarce and studies are very heterogeneous in type of PPs, intervention, dose, and duration of study. Improvement in faecal urgency was seen with curcuminoids nanomicelles [39] and extra virgin olive oil [47]. Lack of abdominal pain was significantly associated with higher dietary daidzein and total isoflavones; however, there was no difference in abdominal pain with higher dietary proanthocyanidins [66]. Lack of constipation was significantly associated with lower dietary glycitein [66] and higher dietary proanthocyaninidins were significantly associated with constipation [65]. Extra virgin olive oil administration resulted in a significant reduction in reports of constipation [47]. One study explored individual gastrointestinal symptoms in CD and found supplementation with Theracurmin significantly reduced stool frequency and demonstrated a trend in reduced abdominal pain [54].

A total of 11 studies investigated effects of PPs on IBS symptoms (Table 3 and Table 4), including tea (*n* = 2), coffee (*n* = 2), chocolate (*n* = 1), Pycnogenol (*n* = 1), soy isoflavones (*n* = 1), soy isoflavones combined with vitamin D (*n* = 1), Crofelemer (*n* = 2), curcumin and fennel essential oil (*n* = 1), ginger (*n* = 1), polydatin combined with palmithoylethanolamide (*n* = 1), and Gelsectan (*n* = 1). The irritable bowel syndrome severity scoring system (IBS-SSS) is a five-item questionnaire that explores questions about gastrointestinal symptoms and the interference of IBS with quality of life [82]. Forty mg of soy isoflavones daily for 6 weeks did not impact IBS-SSS [59]; however, when combined with vitamin D 50,000 IU biweekly, it resulted in a significantly lower score [57,58]. Eighty-four mg curcumin supplement in combination with fennel essential oil for 30 days [62] and 1000 mg ginger for 28 days [64] also significantly reduced IBS-SSS. Although 1000 mg ginger supplementation improved IBS-SSS, there was also improvement in the placebo group [64]. The PP content of the ginger supplement was not available, which limits the ability to interpret the data. Soy isoflavones supplementation also reduced IBS score when assessed using a visual analogue scale (VAS) [59].

There have been positive results from studies suggesting PPs can improve abdominal pain and distention in IBS, but no improvements in stool consistency, frequency, urgency to open bowels, and adequate relief. Pycnogenol is a maritime pine bark extract supplement that contains phenolic monomers, condensed flavonoids, and phenolic acids [83]. Pycnogenol decreased pain on manual abdominal pressure, increased relief of distention and abdominal bowel movement, and reduced the need for rescue medication, although had no effect on frequency of painful attacks [55]. Abdominal pain severity was reduced with supplementation of polydatin combined palmithoylethanolamide [56]. Soy isoflavones combined with vitamin D 50,000 IU biweekly reduced duration of abdominal pain but did not alter abdominal distention [57]. Crofelemer is an oligomeric proanthocyanidin extracted from the bark of *Croton lechleri* [84], and 500 mg/day increased pain and discomfort free days in female subjects [60]. Conversely, crofelemer given at lower doses (125 mg and 250 mg) and in male subjects did not show any difference in pain and discomfort free days [60,61]. No difference was found in stool consistency or frequency or urgency to open bowels with supplementation of any dose of crofelemer [60,61]. Gelsectan is a supplement that combines tannins with xyloglucan, pea protein. Gelsectan supplementation was found to increase frequency of stool type 3–4 on the Bristol stool scale; however, the dose of tannins in Gelsectan was not specified, and therefore the results should be interpreted with caution [63]. Subjects supplemented with ginger reported no difference in adequate relief when opening bowels [64]. In one observational study, 39% of subjects reported coffee consumption was associated with IBS symptoms, while chocolate consumption was associated with IBS symptoms in 28% of the subjects [74]. This study consisted of a small sample size of 330 subjects and should be interpreted with caution, as self reported food symptoms were retrospectively reported, higher in subjects with anxiety, and PP content was not reported.

Few studies have investigated the effect of PPs on IBS compared to IBD. Soy isoflavones and ginger supplementation have been equivocal with regards to IBS-SSS. Curcumin has been most frequently studied across IBD and IBS, particularly in UC. In UC oral curcumin, curcumin enema, curcumin combined with green tea and curcumoids nanomiclles resulted in reduction in disease activity index score (CAI, partial Mayo score, UCDAI, and SSCAI) and induced remission in some instances. The data is scarce for the use of curcumin in CD and IBS. From the available studies, curcumin was found to reduce CDAI in CD, and in combination with fennel essential oil it was also reported to reduce IBS-SSS. The evidence suggests abdominal pain is the main symptom altered by PP supplementation, although there is a lack of consistency with regards to other symptoms of IBS. Crofelemer and Pycnogenol, both bark extracts, are promising supplements for symptom improvement in IBS and warrant further research. Further work in the field of other PPs and symptoms in IBD and IBS is necessary as very few studies have been conducted which limits the ability to form generalisable conclusions.

### 2.2. (Poly)phenols and Inflammatory Markers in Inflammatory Bowel Disease and Irritable Bowel Syndrome

A total of 10 studies investigated the effects of PPs on inflammatory markers in IBD (Table 1, Table 2 and Table 4), including faecal calprotectin, C-reactive protein (CRP), ESR, transforming growth factor beta (TGF-β), tumour necrosis factor-alpha (TNF-α), interferon-gamma (IFN-γ), nuclear factor-kappa beta (NF-κB), (interleukin 8) IL-8, growth regulated oncogene (GRO), granulocyte macrophage colony-stimulating factor (GM-CSF), platelet level, and mean platelet volume.

The effects of a spectrum of PPs have been investigated in relation to inflammatory markers in IBD. The most commonly investigated was ESR, which has been shown to give reliable information on disease activity in IBD [85]. Statistically significant reductions in ESR were found with administration of 1500 mg and 1650 mg curcumin [40,43], 140 mg silymarin [49], 30 g flaxseed, 10 g flaxseed oil [46], and 50 mL extra virgin olive oil [47] in UC. A trend toward reduce ESR was found with 1000 mg curcumin combined with 500 mg green tea [36]. Kedia found no difference in ESR with the administration of 450 mg curcumin per day for 8 weeks in UC [41]. A mean reduction of 10 mm/hr was reported in all 4 subjects with CD supplemented with 1650 mg curcumin [43]. Shapira and Sadeghi used higher doses of curcumin compared to Kedia, which could explain the positive effect on ESR those studies demonstrated.

CRP is an acute phase protein that indicated inflammation and is upregulated on stimulation of interleukin 6 (IL-6), TNF-α, and interleukin 1-beta (IL-1-β) [86]. CRP can be used as an indicator of disease activity in CD and is associated with a strong CRP response, whereas UC is associated with a modest to absent response despite active inflammation [86]. A non-significant reduction in CRP was produced by combining 1000 mg curcumin and 500 mg green tea supplementation in UC [36]. Pure trans-resveratrol (500 mg) [50] and 50 mL extra virgin olive oil [47] resulted in a significant reduction in high sensitivity CRP (hs-CRP) in UC. However, there were no significant changes in CRP with the administration of 840 mg bilberry anthocyanins for 6 weeks in UC [44] or moderate red wine consumption (1–3 glasses) for 1 week in UC or CD [53].

NF-κB is a transcription factor that increases production of pro-inflammatory cytokines such as TNF-α and IFN-γ, enzymes, and adhesion molecules [87,88]. TGF-β is an anti-inflammatory mediator that has a protective role in IBD [89]. A significant reduction in NF-κB p65 and TNF-α was demonstrated with supplementation with pure 500 mg trans-resveratrol in UC [51]. Conversely, there was no change in TNF-α with supplementation of 1500 mg curcumin [40] or 50 mL extra virgin olive oil [47] in UC and 200–400 g mango consumption in UC and CD [52]. Thirty g flaxseed and 10 g flaxseed oil also significantly reduced IFN-γ and increased TGF-β [46].

Blood platelet level and mean platelet volume are indications of platelet number and size of platelets respectively. In IBD, chronic inflammation enhances blood platelet number and larger platelets have increased metabolic and enzymatic activity and play a role in inflammation. [90]. Reduced platelet level and increased mean platelet volume may indicate reduction in disease activity in IBD [91]. One study on curcumin supplementation in UC noted improved platelet level and mean platelet volume with 1500 mg curcumin supplementation for 8 weeks [40].

Calprotectin is a protein found in immune cells and faecal calprotectin is used to indicate disease activity in IBD [92]. Faecal calprotectin was significantly reduced with red wine consumption (1–3 glasses) in subjects with UC and CD [53]; 840 mg bilberry anthocyanin supplementation also reduced faecal calprotectin in subjects with UC, although it had no effect on serum markers associated with inflammation [44].

The mucosa-associated microbiome is a non-invasive source of biomarkers for gut inflammation [93] and may protect the host by production of short chain fatty acids [94]. In CD, 200–400 g mango consumption significantly increased faecal butyric acid and *Lactobacillus* levels, notably *Lactobacillus plantarum*, *Lactobacillus lactis*, and *Lactobacillus reuteri* [52]. In conjunction with lower production of IL-8, GRO, and GM-CSF, this suggests mango consumption reduces neutrophilic induced inflammation in CD [52]

A recent review suggests mucosal inflammation and neuro-inflammation could be involved in the pathophysiology of IBS [95]. In line with a limited but emerging understanding of inflammatory changes in IBS, there has been investigation into the inflammatory response in IBS following PP administration, with only two studies exploring inflammatory markers. Consumption of 40 mg soy isoflavones results in a significant reduction in TNF-α and lower levels of NF-κB [58], but supplementation of 20 mg polydatin combined with 200 mg palmitoylethanolamide supplementation did not affect mast cell count over time [56].

ESR is the inflammatory marker with the most consistent evidence relating to the impact of PPs on inflammation in IBD. Curcumin, silymarin, flaxseed and flaxseed oil, and extra virgin olive oil resulted in significant reductions in ESR, although lower dose curcumin and curcumin combined with green tea did not produce the same results. The nature of the impact of PPs on other inflammatory markers remains unclear and further work is needed considering faecal calprotectin. In two studies, hs-CRP was positively associated with pure trans-resveratrol and extra virgin olive oil supplementation UC; however, association with CRP in UC and CD were inconsistent. Limited work on inflammatory marker has been completed in IBS (Table 3). From the two studies included in this review, it is difficult to draw conclusions but warrants further work. Only one study explored mucosa-associated microbiota and neutrophilic induced inflammation with mango consumption, which is an exciting area for further research.

### 2.3. (Poly)phenols and Health-Related Quality of Life in Inflammatory Bowel Disease and Irritable Bowel Syndrome

A total of six studies investigated the effects of PPs on health-related quality of life in IBD and three studies in IBS (Table 1, Table 2 and Table 3). The inflammatory bowel disease questionnaires (IBDQ, IBDQ-9), short inflammatory bowel disease questionnaire (SIBDQ), IBS quality of life score (IBS-QOL) and EQ-5D-3L score were standardised questionnaires used to explore this outcome measure.

The inflammatory bowel disease questionnaire (IBDQ) is a 32 item health related quality of life questionnaire validated for use in IBD [96]. The IBDQ has subsequently been reduced to 10 item (short inflammatory bowel disease questionnaire, SIBDQ) [97] and 9 item (IBDQ-9) [98] questionnaires. IBDQ and SIBDQ include the following four domains of functioning and well-being: bowel symptoms, systemic symptoms, emotional function, and social function [96,97]. IBDQ is the most widely used health-related quality of life tool in IBD and both the shorter SIBDQ and IBDQ-9 correlate well with it [99]. IBDQ, IBDQ-9, and SIBDQ have been used in studies to assess the benefits of PPs on IBD.

All PPs investigated that explored IBDQ and IBDQ-9 as an outcome measure reported an increased score in UC. Both 200 mg ginger powder [48] and 400–800 mg green tea EGCG [45] significantly increased IBDQ in participants with UC, while 1500 mg curcumin [40], 30 g flaxseed, 10 g flaxseed oil [46], and 500 mg pure trans-resveratrol [50,51] resulted in significant increases in IBDQ-9 in participants with UC. Curcuminoids nanomicelles also increased wellbeing score [39]. Mango consumption (200–400 g) had no effect on SIBDQ in UC or CD [52].

The irritable bowel syndrome quality of life questionnaire (IBS-QOL) is a health related quality of life measure specific to IBS consisting of 41 items [100]. A limited number of studies have reported using the IBS-QOL in PP interventions. Soy isoflavones (40 mg) significantly increased IBS-QOL [57,59], 84 mg curcumin in combination with 50 mg fennel essential oil [62] and two capsules of Gelsectan [63] resulted in a non-significant increase in IBS-QOL. Gelsectan also results in a non-significant improvement in general health-related quality of life measure EQ-5D-3L score [63].

Health-related quality of life remains an under investigated outcome on the supplementation of PPs in IBD and IBS. Limited evidence suggests that health-related quality of life can be improved by PP supplementation in UC although more work is required in CD and IBS.

### 2.4. Habitual (Poly)phenol Consumption on Incidence of Inflammatory Bowel and Irritable Bowel Syndrome

A total of three case-control studies investigated habitual PP consumption on incidence of IBD, including one study on tea and coffee intake, one study on general PPs, and one study on isoflavones (Table 4). The results presented here need to be interpreted with caution, as individuals with IBD did not exceed 256. The studies on isoflavones, tea, and coffee consumption were retrospective and the study on general PPs was prospective.

Using the International Organisation of IBD (IOIBD) questionnaire on environmental factors, one study with 256 individuals with UC and 186 individuals with CD showed a significant association between daily tea consumption and reduced odd of developing both UC and CD (0.630 and 0.662 respectively) [69]. The same study showed contradictory effects with coffee consumption, as it was significantly associated with reduced odds of UC (0.630), but no difference in the odds of CD [69]. Country specific food frequency questionnaires were used to assess PP content of habitual dietary intake using the phenol explorer database adjusting for cooking methods [70]. There was no association with total PP and odds of both UC and CD; however, in the third quartile of flavonols intake, there was a lower odds ratio of UC [70]. Conversely, one study found increased odds of UC, including odds of disease reaching the cecum or ileum, with higher intakes of isoflavones following a diet history questionnaire of isoflavones intake one month and one year before diagnosis [67].

A total of three cross-sectional observational studies have explored the impact of tea and coffee consumption on the odds of developing IBS with contradictory results (Table 4). Consumption of three cups of coffee per day [71] and being a non-coffee drinker [73] have both been associated with increased odds ratios for developing IBS. There has also been evidence to suggest no difference in odds ratio [71] and increased odds ratio [72] with tea consumption for developing IBS.

In nutritional research, it can be a challenge to use retrospective dietary assessment of the distant past as this holds only moderate correlation with reports at the time [101]. With prospective research assuming consistent dietary intake following data gathering is also problematic. Development of IBS and IBD is still not fully understood and multifactorial; therefore, the interplay of other factors requires close attention [8,23]. The PP content of tea and coffee is not explored in the studies; therefore, other dietary components may also affect the odds of developing disease. Small numbers of observational studies have explored the association of PPs and the development of IBD and IBS with contradictory results; therefore, it is difficult to draw firm conclusions on their effect.

### 2.5. (Poly)phenols and Intestinal Permeability in Inflammatory Bowel Disease and Irritable Bowel Syndromee

A total of one prospective cohort study and one uncontrolled study investigated the effects of PPs on intestinal permeability in IBD (Table 1 and Table 2). One RCT study investigated the effects of PPs on intestinal permeability in IBS (Table 3).

Intestinal permeability refers to functionality of the intestinal barrier with impaired permeability resulting in loss of intestinal homeostasis and functional impairment [102]. IBD and IBS are both classified as a disease related to intestinal permeability [102]. The lactulose/mannitol (L/M) ratio is a test of small bowel permeability [103] and urinary sucralose is a test of whole gut permeability [104]. Serine protease activity has recently been associated with intestinal permeability [105] and lipopolysaccharide (LPS) permeability is linked with intestinal and systemic inflammation [52]. Only two studies with red wine and mango investigated intestinal permeability in IBD. One week of moderate red wine consumption (1–3 glasses) disrupted intestinal barrier function in IBD [53]. Red wine did not lower L/M ratio in UC, but significantly increased urinary sucralose excretion, suggesting increased whole gut permeability [53]. In CD, red wine consumption increased L/M ratio, suggesting increased small bowel permeability [53]; 200–400 g mango consumption resulted in a trend towards reduced plasma LPS production [52]. Unfortunately, neither of these studies reported the polyphenol content; therefore; results need to be interpreted with caution. In IBS soy isoflavones, supplementation resulted in a significant reduction in serine protease activity [58].

From the studies presented, soy isoflavones may decrease intestinal permeability in IBS but work in IBD is equivocal. There has been in vitro and in vivo studies on the mechanism and effect of PPs on intestinal permeability; however, research in humans remains in its infancy [106]. PPs and intestinal permeability are being considered in older subjects [107], but a larger body of work is required to increase the evidence base for the effect of PPs on intestinal permeability in IBD and IBS.

### 2.6. (Poly)phenols and Endoscopic Response in Inflammatory Bowel Disease

Mucosal healing has become an important endpoint in IBD therapy [108]. Endoscopic features evaluated in regards to IBD include vascular pattern, mucosal erythema, friability and bleeding, ulceration, and granulation [76,79]. The Mayo score, UCDAI, and DAI all include endoscopic assessment subscores, but only nine studies discuss endoscopic results (six with curcumin, one with curcumin combined with green tea, one with bilberry anthocyanins, and one with green tea EGCG). In UC, 2000 mg curcumin supplementation significantly improved the Rachmilewitz endoscopic index score [37] and endoscopic disease activity defined as a decrease by at least 1 in mucosal appearance score of UCDAI [42] as well as correlating significantly with clinically remission based on endoscopy [38]. Curcumin (1650 mg) also reduced semi-quantitative endoscopic score in 2 out of 5 subjects in one small study [43]. Curcumin (1000 mg) in combination with 500 mg of green tea extract also reduced mean endoscopic Mayo score by 0.68 in UC, but it is not clear whether this effect was on a particular descriptor (e.g., vascular pattern or friability) [36]. Significant reduction in endoscopic Mayo score in participants with UC was associated with bilberry anthocyanin consumption [44]. The endoscopic component of the UCDAI score reduced in subjects with UC who demonstrated an overall reduction in UCDAI following green tea EGCG supplementation [45]. In CD, 360 mg of curcumin (Theracurmin) significantly reduced simple endoscopic score for Crohn’s disease (SESCD) and anal lesions, although there was no difference in endoscopic remission rates defined at SESCD ≤ 4 [54].

From the limited numbers of studies reporting endoscopic response in IBD, there is consistent indication that administration of curcumin alone or in combination with green tea confers small, but definite endoscopic improvements in IBD. Green tea EGCG also improved endoscopic response, which supports findings of the curcumin combined with green tea. Only one small study explored bilberry anthocyanin with positive outcomes, but this should be interpreted with caution. Future studies should consider separately reporting on the endoscopic components of disease activity indices to expand the knowledge and evidence base on the benefits of PPs on endoscopic response in IBD.

### 2.7. (Poly)phenols and Oxidative Stress in Inflammatory Bowel Disease

It has been proposed that inflammatory injury to tissues leads to oxidative stress by increased production of reactive oxygen species [109]. However, whether direct antioxidant capacity of PPs in vivo is relevant to human health has not been established and is still a matter of intense debate [110]. One RCT investigated the antioxidant effects of pure trans-resveratrol in UC (Table 1) [51]. Total antioxidant capacity (TAC), malondialdehyde (MDA), and superoxide dismutase (SOD) were significantly reduced after supplementation with 500 mg pure trans-resveratrol, suggesting increased antioxidant capacity and reduced oxidative stress [51]. The impact of PPs on oxidative stress is an emerging area of research in IBD; however, evidence is lacking and an increased number of high quality studies is need to provide further insight into the potential benefits. Future work will aid understanding of the link between inflammatory injury, oxidative stress and the role that PPs play.

## 3. Limitations and Strengths of the Current Evidence

A strength of the included studies is that many of the interventional studies were well conducted double-blind RCTs. Cross over and factorial study designs have also been implemented to increase sensitivity and efficiently evaluate results. Many of the symptom and health-related quality of life questionnaires were validated and widely used, thus allowing comparison between studies.

A small sample size is the main limitation for many of the included interventional studies, followed by short intervention periods preventing the establishment of long term efficacy data. Endoscopic outcomes are of particular relevance in IBD; however, due to its invasive nature and cost, many studies could not include endoscopic samples or gather complete endoscopic data from participants. Only two studies incorporated a dose-finding study design [45,60], thus reducing the ability to provide clarity of the optimal doses of PPs. Of the studies included, four studies were uncontrolled trials. Of interest, one study removed the fibre supplement placebo control, as participants with IBD were not willing to be randomized due to the perceived side effects of fibre [52]. An important limitation is that many interventional and observational studies included PP containing foods, but failed to provide detail of the PP content; therefore, it is very difficult to draw conclusions from them. In addition, none of the studies reported the bioavailability of the PP in blood or urine, and compliance was not assessed with adequate biomarkers. This is of importance in particular in the curcumin studies, due to its well known low bioavailability.

## 4. Methods

**Figure 1:**
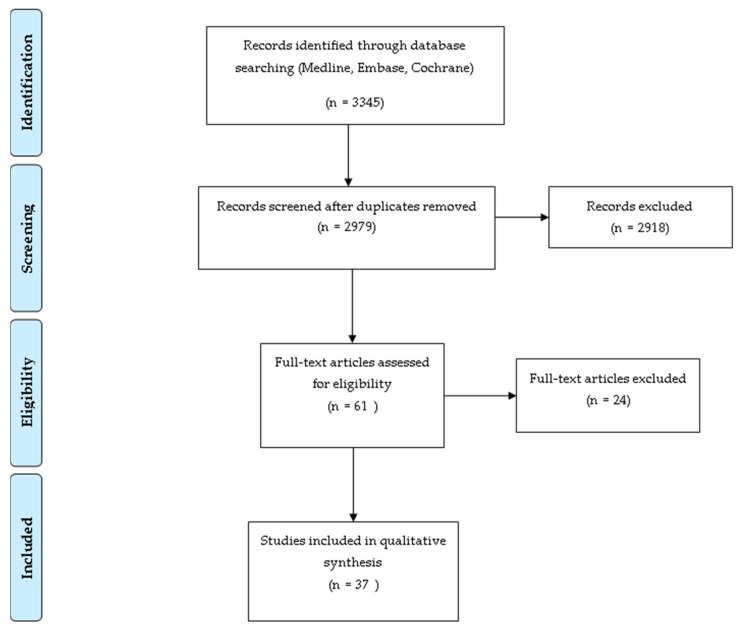
Preferred Reporting Items for Systematic reviews and Meta-Analyses (PRISMA) flow diagram for identification of relevant included and excluded studies.

Studies were identified by searching the electronic databases Medline, EMBASE, and Cochrane up to and including December 2020. Search terms associated with PPs and their derivatives were combined with IBS, CD, UC, and their symptoms (see  Supplementary S1 in Supplementary Materials for the Medline, EMBASE, and Cochrane search strategies). Eligibility assessment was performed in a standardised manner and information was inputted into a data extraction sheet by one reviewer (MH). Intervention and observational studies on adults reported in English were included. Exclusion criteria included study protocols, abstracts without full article, review articles, studies without clear specification of PPs, or PP containing foods and studies on other gastrointestinal conditions apart from IBS and IBD. A total of 37 studies were eligible for inclusion, 13 studies in IBS, and 24 studies in IBD as shown in the Preferred Reporting Items for Systematic reviews and Meta-Analyses (PRISMA) flow diagram (Figure 1). Information extracted from each study included: characteristics of participants, type of intervention, and results.

## 5. Conclusions

A range of PPs have been investigated in relation to gastrointestinal disorders. PPs have been used as adjuvant therapy with some positive outcomes. Curcumin has received the most attention, with 11 studies exploring its benefits. Of note, a recent technical review on curcumin in UC classified the studies included as low quality due to risk of bias, inconsistencies, and imprecision [111]. It is difficult to form firm conclusions on the benefits of PPs as more large scale interventional studies are required, particularly in IBD, where participant numbers have not exceeded 100. From the current evidence base, there is promise that symptomatic and quality of life improvements can be found from supplementation with PPs. In UC, oral curcumin, curcumin enema, curcumin combined with green tea, green tea EGCG, bilberry anthocyanins, flaxseeds, flaxseed oil, and silymarin resulted in positive improvement in disease activity index (UCDAI, Mayo score, DAI). Limited work has explored health-related quality of life in IBD and IBS, but there is promising work with regards to IBDQ and IBDQ health-related quality of life measures. Improvement in IBDQ and IBDQ-9 was seen in all PPs studies that reported these outcomes (ginger, curcumin, green tea EGCG, pure trans-resveratrol, flaxseeds, and flaxseed oil). Incorporating health-related quality of life measures into the experimental design of interventional studies would provide a valuable addition to the evidence base.

Reported endoscopic findings consistently highlight that benefits have been seen in IBD from supplementation with PPs, particularly with curcumin supplementation; therefore, curcumin supplementation warrants further large scale interventional trials to confirm its benefits. Development of IBD and IBS can be multifactorial therefore observational studies on the odds of developing IBD and IBS based on habitual dietary intake needs to be interpreted with caution. Dietary intakes of PPs tend to be lower than the PP doses used in interventional studies; therefore, it can be difficult to draw conclusions about the role dietary PPs play in IBD and IBS. However, interventional studies did not report habitual dietary intake, which could have a combined effect with the intervention PP supplement.

The evidence base for the impact of PPs on inflammatory markers in IBD and IBS remains equivocal and requires further investigation. However, ESR is an inflammatory marker with the strongest link with PPs and IBD from the evidence reviewed. More recent work has explored intestinal permeability and PPs in IBD and IBS. Soy isoflavones reduced serine protease activity, suggesting an improvement in intestinal permeability in IBS. In IBD, moderate red wine consumption increased intestinal permeability and mango consumption decreased intestinal permeability. In IBD, both studies compared whole foods and not PPs alone; therefore, further work is needed to establish a relationship between PPs and intestinal permeability. Implications of PPs on oxidative stress in IBD and IBS also requires further work, as one study exploring the impact of pure trans-resveratrol yielded interesting findings on increasing antioxidant capacity in UC. Intestinal enzymes and colonic microflora play a role in the absorption of PPs; therefore, bioavailability of PPs needs to be considered [112]. Curcuminoids nanomicelles and Theracurmin, which are preparations of curcumin with enhanced bioavailability, produced positive results at lower doses than standard curcumin preparations. Consideration of intestinal enzyme activity and microbiome as part of research studies would provide valuable mechanistic insight in vivo.

In conclusion, promising work has been undertaken with regards to PPs and IBD and IBS, but to clarify the impact of PPs further, long term trials that encompass clinical and mechanistic work are required.

## Figures and Tables

**Table 1:** Summary of human intervention studies of (poly)phenols in ulcerative colitis (UC).

Reference	Subject Description	Product	Dosage/Duration of Intake	Main Findings
Shapira et al. [36]	16 men and women with active mild-to-moderate UC	Curcumin and green tea	1000 mg curcumin, 500 mg green tea/8 weeks	- 4.2 point improvement in mean CAI score **
				- Reduction in CRP and ESR
				- 0.68 reduction in mean endoscopic Mayo score *
Hanai et al. [37]	83 men and women with UC with confirmed remission	Curcumin	Control dose 0 mg, curcumin 2000 mg/6 months	- Improvement in mean CAI score *
				- Improvement in Rachmilewitz endoscopic index score **
Lang et al. [38]	50 men and women with mild-to-moderate UC	Curcumin	Control dose 0 mg, Curcumin 3000 mg/1 month	- Reduction of ≥1 in partial Mayo score **
				- 56% of subjects achieved a SCCAI score ≤ 2 **
				- Clinical remission based on endoscopy *
				- Reduction of ≥3 points in SCCAI score **
Masoodi et al. [39]	56 men and women with mild-to-moderate UC	Curcuminoids nanomicelles	Control dose 0 mg, curcuminoids 240 mg/4 weeks	- No difference in frequency of defecation or blood in stools
				- Reduction in faecal urgency *
				- Reduction in mean SCCAI score *
				- Improvement in wellbeing score *
Sadeghi et al. [40]	70 men and women with mild-to-moderate UC	Curcumin	Control dose 0 mg, 1500 mg curcumin/8 weeks	- Reduction of ≥3 points in SCCAI score **
				- 83.9% of subjects achieved SCCAI score ≤ 2 **
				- 27% increase in mean IBDQ-9 score **
				- Decrease in ESR * and serum hs-CRP *
				- Improvement in platelet level ** and mean platelet volume *
				- No change in TNF-α
Kedia et al. [41]	41 men and women with mild-to-moderate UC	Curcumin	Control dose 0 mg, 450 mg curcumin/8 weeks	- No difference in UCDAI or mucosal healing
				- No difference in ESR
Singla et al. [42]	45 men and women with mild-moderate UC	Curcumin	Control dose 0 mg, 140 mg Curcumin enema/8 weeks	- Reduction in UCDAI score by ≥3 **
				- UCDAI score ≤ 3 *
				- Decrease in mucosal appearance score of UCDAI by ≥1 *
Holt et al. [43]	5 men and women with UC	Curcumin	1100 mg curcumin/1 month then 1650 mg/1 month	- Improvement in UC judged by global score *
				- Reduction of endoscopic score in 40% of subjects
Biedermann et al. [44]	11 men and women with active mild-to-moderate UC	Bilberry-anthocyanin	Control dose 0 g, 160 g bilberry corresponding to 840 mg anthocyanin/6 weeks	- Improvement in mean CAI score *
				- Reduction in the endoscopic Mayo score * and complete Mayo score **
				- Reduction in faecal calprotectin *
				- No relevant changes in serum markers of inflammation
Dryden et al. [45]	15 men and women with mild-to-moderate UC	Green tea polyphenols-EGCG	Control dose 0 mg, cohort 1400 mg, cohort 2800 mg EGCG/8 weeks	- Reduction in UCDAI score *
				- 53% of subjects achieved a UCDAI score < 2
				- Increase in IBDQ score
				- Subjects with reduction in UCDAI score exhibited a reduction in their endoscopic score
Morshedzadeh et al. [46]	75 men and women with mild-to-moderate UC	Flaxseed or Flaxseed oil	Control dose 0 g, 30 g ground flaxseed, or 10 g flaxseed oil/12 weeks	- Reduction in the serum levels of ESR **, IFN-γ ** and IL-6 **
				- Increase in the TGF-β * and IBDQ-9 score **
				- Reduction in faecal calprotectin ** and Mayo score **
Morvaridi et al. [47]	32 men and women with mild-to-moderate or in remission UC	Extra virgin olive oil	Control dose 50 mL canola oil, 50 mL extra virgin olive oil/20 days	- Reduction in ESR *, hs-CRP **
				- No change in TNF-α
				- No change in Mayo score
				- Reduction in bloating *, constipation **, faecal urgency **, and incomplete defecation *
				-Reduction in GSRS **
Nikkhah-Bodaghi et al. [48]	46 men and women with mild-to-moderate UC	Ginger powder	Control dose 0 mg, 200 mg ginger powder/12 weeks	- Reduced MDA **
				- Improved SCCAI and IBD-Q *
				- No difference in TAC total antioxidant capacity
Rastegarpanah et al. [49]	80 men and women with UC in remission	Silymarin	Control dose 0 mg, 140 mg silymarin/6 months	- Improvement in ESR *, DAI *
Samsami-kor et al. [50,51]	49 men and women with mild-to-moderate UC	Pure trans-resveratrol	Control dose 0 mg, 500 mg pure trans-resveratrol/6 weeks	- Reduction in hs-CRP **, TNF-α **, NF-κB p65 **, MDA **, TAC **, and SOD **
				- Increase in IBDQ-9 **
				- Decreased in SCCAI **
Kim et al. [52]	7 men and women with UC	Mango	200–400 g mango pulp corresponding to 95.18–190.36 mg of pro-gallic acid/8 weeks	- No effect on SIBDQ or TNF-α
				- Decreased in SCCAI *
				- Lower production of IL-8, GRO, GM-CSF * LPS *
				- Increased *Lactobacillus* * (*L.plantarum* **, *L.lactis* ** and *L.reuteri* *) and butyric acid *
Swanson et al. [53]	8 men and women with UC in remission	Red wine	1–3 glasses red wine/1 week	- No change in CRP, UCAI, L/M ratio
				- Reduction in faecal calprotectin **
				- Increased urinary sucralose excretion *

Ulcerative colitis (UC); clinical activity index (CAI); C-reactive protein (CRP); erythrocyte sedimentation rate (ESR); epigallocatechin-3-gallate (EGCG); ulcerative colitis disease activity index (UCDAI); inflammatory bowel disease questionnaire (IBDQ); simple clinical colitis activity index (SCCAI); tumour necrosis factor-alpha (TNF-α); interferon-gamma (IFN-γ); transforming growth factor-beta (TGF-β); interleukin 6 (IL-6); high sensitive CRP (hs-CRP); nuclear factor-kappa beta (NF-κB); gastrointestinal symptom rating scale (GSRS); malondialdehyde (MDA); total antioxidant capacity (TAC); superoxide dismutase (SOD); disease activity index (DAI); short inflammatory bowel disease questionnaire (SIBDQ); interleukin 8 (IL-8); growth regulated oncogene (GRO); granulocyte macrophage colony-stimulating factor (GM-CSF); lipopolysaccharide (LPS); ulcerative colitis clinical activity index (UCAI); lactulose/mannitol (L/M); * *p* ≤ 0.05; ** *p* ≤ 0.01.

**Table 2:** Summary of human intervention studies of (poly)phenols in Crohn’s disease (CD).

Reference	Subject Description	Product	Dosage/Duration of Intake	Main Findings
Holt et al. [43]	Four men and women with CD	Curcumin	1080 mg curcumin/1 months then 5760 mg/2 months	- Reduction in CDAI and ESR
Sugimoto et al. [54]	17 men and women with mil-to-moderate CD	Theracurmin (curcumin)	Control dose 0 mg, 360 mg Theracurmin/12 weeks	- Reduction in CDAI *, SESCD * stool frequency * and abdominal pain
				- Reduction in anal lesions *
				- No difference in CRP, SESCD ≤ 4
Swanson et al. [53]	Six men and women with CD	Red wine	1–3 glasses red wine/1 week	- No change in CDAI, CRP, urinary sucralose
				- Reduction in faecal calprotectin **
				- Increase in L/M ratio *
Kim et al. [52]	Three men and women with CD	Mango	200–400 g mango pulp/8 weeks	- No effect on SIBDQ or TNF-α
				- Decreased in SCCAI *
				- Lower production of IL-8 *, GRO *, GM-CSF * LPS *
				- Increased *Lactobacillus* * (*L.plantarum* **, *L.lactis* ** and *L.reuteri* *) and butyric acid *

Crohn’s disease (CD); Crohn’s disease activity index (CDAI); erythrocyte sedimentation rate (ESR); simple endoscopic score for Crohn’s disease (SESCD); short inflammatory bowel disease questionnaire (SIBDQ); simple clinical colitis activity index (SCCAI); interleukin 8 (IL-8); growth regulated oncogene (GRO); granulocyte macrophage colony-stimulating factor (GM-CSF); lipopolysaccharide (LPS); * *p* ≤ 0.05; ** *p* ≤ 0.01.

**Table 3:** Summary of human intervention studies of (poly)phenols in irritable bowel syndrome (IBS).

Reference	Subject Description	Product	Dosage/Duration of Intake	Main Findings
Belcaro et al. [55]	77 men and women IBS	Pycnogenol (Maritime pine bark)	Control dose 0 mg, 150 mg Pycnogenol/4 weeks	- No difference in painful attacks
				- Decrease in mild pain on manual abdominal pressure *
				- Relief of distension/abdominal bowel movements *
				- Reduced use of rescue medication *
Cremon et al. [56]	54 men and women with IBS	Palmithoylethanolamide/polydatin	Control dose 0 mg, 200 mg palmithoylethanolamide, 20 mg polydatin/12 weeks	- No difference in mast cell count over time
				- Improved abdominal pain severity *
Jalili et al. [57,58]	100 women with IBS	Soy isoflavones (with vitamin D)	Control dose 0 mg, 20 mg of daidzein, 17 mg of genistein, and 3 mg of glycitein/6 weeks	- Lower IBS-SSS **
				- Reduction in TNF-α ** and abdominal pain duration ** and life disruption **
				- Lower NF-κB ** and serine protease activity **
				- Increased satisfaction of bowel habits –Improved IBS-QOL **
				- No difference in abdominal distention
Jalili et al. [59]	45 women with IBS	Soy isoflavones	Control dose 0 mg, 20 mg of daidzein, 17 mg of genistein, and 3 mg of glycitein/6 weeks	- No difference in IBS-SSS
				- Improved IBS-QOL score **
				- Reduction in score of IBS as VAS **
Mangel et al. [60]	241 men and women with IBS	Crofelemer (oligomeric proanthocyanidins)	Control dose 0 mg, 125 mg, 250 mg, or 500 mg proanthocyanidins (crofelemer)/12 weeks	- No difference in stool consistency or frequency, faecal urgency, or adequate relief
				- Increase in pain and discomfort free days in female subjects with 500 mg crofelemer **
Nee et al. [61]	237 women with IBS	Crofelemer (oligomeric proanthocyanidins)	Control dose 0 mg, 250 mg proanthocyanidins (crofelemer)/12 weeks	- No difference in pain/discomfort free days, pain/discomfort score, stools consistency or frequency, faecal urgency, or adequate relief
Portincasa et al. [62]	116 men and women with IBS	Curcumin and fennel essential oil	Control dose 0 mg, 84 mg curcumin, 50 mg fennel essential oil/30 days	- Decrease in mean IBS-SSS **
				- Increase in IBS-QOL score
Trifan et al. [63]	60 men and women with IBS	Gelsectan (Tannins, xyloglucan, pea protein)	Control dose 2 placebo capsules, 2 capsules/4 weeks	- Increase in Bristol stool form scale type 3–4 **
				- Improvement in abdominal pain, bloating, IBS-QOL score, and EQ-5D-3L score
Van Tilberg et al. [64]	44 men and women with IBS	Ginger	Control dose 0 mg, 1000 mg, or 2000 mg ginger/4 weeks	- Reduction in IBS-SSS with placebo and 1 g ginger **
				- No difference in reports of adequate relief

Irritable bowel syndrome (IBS); IBS severity scoring system (IBS-SSS); tumour necrosis factor-alpha (TNF-α); nuclear factor-kappa beta (NF-κB); IBS quality of life questionnaire (IBS-QOL); visual analogue scale (VAS); EQ-5D-3L score-health related quality of life score; * *p* ≤ 0.05; ** *p* ≤ 0.01.

**Table 4:** Summary of observational studies of (poly)phenols in inflammatory bowel disease (IBD) and irritable bowel syndrome (IBS).

Reference	Study Population	Type of PP/PP Food	Dietary Intake Assessment	Main Findings
Glabska et al. [65]	55 men and women with UC with confirmed remission	Proanthocyanidins	3 day self-reported dietary record	- No difference with abdominal pain, presence of blood or pus in stool, flatulence, or tenesmus
				- Increase mucus in stool and constipation with higher proanthocyanidin intake *
Glabska et al. [66]	56 men and women with UC with confirmed remission	Isoflavones	3 day self-reported dietary record	- Correlation between higher intakes of daidzein ** and total isoflavones * with lack of abdominal pain
				- No correlation between isoflavones intake and bowel movements, flatulence, or tenesmus
				- Correlation between lower glycitein intake and lack of constipation *
Ohfuji et al. [67]	126 men and women with new diagnosed UC	Isoflavones	Self-administered diet history questionnaire—1 month of habits	- Increased OR for UC in highest tertile of isoflavone, daidzein, and genistein intake *
				- Increased OR for disease reaching the cecum or ileum *
Skolmowska et al. [68]	56 men and women with UC in remission	Isoflavones	3 day self-reported dietary record	- No difference in presence of faecal blood
				- Higher intake of daidzein and total isoflavones associated with lack of faecal mucus
				- Higher intake of daidzein associated with lack of faecal pus
Ng et al. [69]	186 men and women with CD, 256 men and women with UC	Tea and coffee	International organisation of IBD environmental factor questionnaire—food habits	- Daily tea and coffee reduced odds of UC **
				- No difference in odds of CD with daily coffee intake
				- Tea reduced odds of CD *
Lu et al. [70]	110 men and women with CD, 244 men and women with UC	(Poly)phenols	Food frequency questionnaire	- Lower OR for UC in third quartile of flavonols intake *
				- Reduced OR for CD with increased flavones and resveratrol intake *
				- No association with total polyphenols and odds of CD or UC
Al Saadi et al. [71]	302 men and women	Tea and coffee	Questionnaire—lifestyle factors	- Increased OR for IBS with consumption of 3 cups of coffee per day *
				- No difference in odds of IBS with tea intake
Ligaarden et al. [72]	388 men and women	Tea	Food frequency questionnaire	- Increased OR for IBS with consumption of 100 mL tea per day *
Siah et al. [73]	297 men and women	Coffee	Dietary questionnaire	- Increased OR for IBS in non-coffee drinkers **
Simren et al. [74]	330 men and women with IBS	Coffee and chocolate	Food questionnaire	- 39% of subjects reported coffee intake produced symptoms of IBS
				- 28% of subjects reported chocolate intake produced symptoms of IBS

Ulcerative colitis (UC); Crohn’s disease (CD); irritable bowel syndrome (IBS); odds ratio (OR); * *p* ≤ 0.05; ** *p* ≤ 0.01.

## Supplementary Materials

The following are available online. Supplementary S1: Database search strategies.
