# Supplementary material for: (Poly)phenols in Inflammatory Bowel Disease and Irritable Bowel Syndrome: A Review
Source: Molecules. 2021 Mar 25;26(7):1843. doi: 10.3390/molecules26071843 (PMC8036772; doi:10.3390/molecules26071843)
Supplement: Supplementary file 1 [file molecules-26-01843-s001.pdf]

Supplementary 1S. Database search strategies

Search strategy: EMBASE

01. exp "CROHN DISEASE"/
02. ((crohn OR crohn's OR crohns) ADJ2 (disease* OR colitis)).ti,ab
03. ((ileitis OR enteritis) ADJ2 (terminal OR regional)).ti,ab
04. ((colitis OR enteritis) ADJ2 granuloma*).ti,ab
05. (ileocoli*).ti,ab
06. (epithelioid ADJ2 granuloma*).ti,ab
07. (inflamm* ADJ2 bowel).ti,ab
08. exp "ULCERATIVE COLITIS"/
09. exp PROCTOCOLITIS/
10. exp PROCTITIS/
11. (inflamm* ADJ2 (colon* OR bowel)).ti,ab
12. (ulcer* ADJ colitis).ti,ab
13. (pancolitis OR rectitis OR proctocolitis OR procto-colitis OR colorectitis OR rectocolitis OR rectocolitis OR recto-sigmoiditis OR rectosigmoiditis OR procto-sigmoiditis OR proctosigmoiditis OR proctitis).ti,ab
14. ((total OR sub-total OR subtotal OR extensive OR left-sided OR universal) ADJ colitis).ti,ab
15. exp POLYPHENOL/
16. exp FLAVONOID/
17. exp LIGNAN/
18. exp STILBENE/
19. exp ANTHOCYANIN/
20. (Polyphenol* OR Phytochemical* OR Phenol* OR Flavonoid* OR Anthocyanin* OR Flavanol* OR Flavanones OR Flavon* OR Flavonol* OR Isoflavone* OR Proanthocyanidin* OR Procyanidin* OR "Phenolic acid*" OR "Hydroxycinnamic acid*" OR "Hydroxybenzoic acid" OR Lignan* OR Stilbene* OR Chalcone* OR Dihydrochalcone* OR Dihydroflavanols OR Isoflavanoids OR Alkylphenols OR Hydroxybenzaldehyde* OR Hydroxycoumarin* OR "Hydroxyphenylacetic acid*" OR "Hydroxyphenylpropanoic acid*" OR "Hydroxyphenylpentanoic acid*" OR Methoxyphenol* OR Tyrosol* OR Elagitannin* OR "Coumaric acid*" OR "Gallic acid*" OR Phytoestrogen* OR Catechin* OR Epicatechin* OR Epigallocatechin* OR Flavan-3-ol OR Gallocatechin* OR Gallate* OR Anthocyanidin* OR Theaflavin* OR Thearubigin* OR "Hydrolyzable Tannin*" OR "Ellagic Acid*").ti,ab
21. (tea OR "pu-er tea" OR "pu-erh tea" OR "Oolong tea" OR "black tea" OR "green tea" OR "red wine" OR grape OR grapeseed OR blueberr* OR cranberr* OR strawberr* OR blackberr* OR raspberr* OR blackcurrant OR berry OR cocoa OR chocolate OR coffee OR apple OR cider OR beer OR orange OR citrus OR grapefruit OR mango OR soy* OR "olive oil" OR flaxseed OR walnut* OR nuts OR almond OR pomegranate OR plum OR cherry OR potato OR sweet potato OR beans OR lettuce OR cabbage OR broccolis OR onion OR botanicals OR rosemary OR sage OR oregano OR clove OR cinnamon OR curry* OR coriander OR parsley OR thyme OR ginger).ti,ab
22. (poly ADJ2 phenol*).ti,ab
23. ((intestin* OR gastrointestin* OR colon* OR bowel*) ADJ2 (motility OR sensitiv* OR functional OR irritable OR irritat* OR gas* OR spastic* OR unstable OR instability OR spasm*)).ti,ab
24. (flatus).ti,ab

25. (wind).ti,ab
26. exp WEATHER/
27. 25 not 26
28. (bloat*).ti,ab
29. (irritable bowel syndrome).ti,ab
30 (IBS).ti,ab
31. ((faecal OR fecal) ADJ2 incontinen*).ti,ab
32. exp DYSPEPSIA/
33. exp "GASTROINTESTINAL MOTILITY"/
34. exp FLATULENCE/
35. exp "IRRITABLE COLON"/
36. exp "FECES INCONTINENCE"/
37. (23 OR 24 OR 27 OR 28 OR 29 OR 30 OR 31 OR 32 OR 33 OR 34 OR 35 OR 36)
38. (diarrhoea OR diarrhea).ti,ab
39. exp DIARRHEA/
40. (constipat*).ti,ab
41. exp CONSTIPATION/
42. (38 OR 39 OR 40 OR 41)
43. (37 OR 42)
44. (1 OR 2 OR 3 OR 4 OR 5 OR 6 OR 7)
45. (8 OR 9 OR 10 OR 11 OR 12 OR 13 OR 14)
46. (15 OR 16 OR 17 OR 18 OR 19 OR 20 OR 21 OR 22)
47. (43 AND 46)
48. (44 AND 46)
49. (45 AND 46)
50. 47 [Languages English] [Human age groups Adult 18 to 64 years OR Aged 65+ years] [Humans] [Records from Embase]
51. 48 [Languages English] [Human age groups Adult 18 to 64 years OR Aged 65+ years] [Humans] [Records from Embase]
52. 49 [Languages English] [Human age groups Adult 18 to 64 years OR Aged 65+ years] [Humans] [Records from Embase]

Search strategy: Medline
01. exp "CROHN DISEASE"/
02. ((crohn OR crohn's OR crohns) ADJ2 (disease* OR colitis)).ti,ab
03. ((ileitis OR enteritis) ADJ2 (terminal OR regional)).ti,ab
04. ((colitis OR enteritis) ADJ2 granuloma*).ti,ab
05. (ileocoli*).ti,ab
06. (epithelioid ADJ2 granuloma*).ti,ab
07. exp "INFLAMMATORY BOWEL DISEASES"/
08. (inflamm* ADJ2 bowel).ti,ab
09. (1 OR 2 OR 3 OR 4 OR 5 OR 6 OR 7 OR 8)
10. (colitis, ulcerative).ti,ab
11. exp PROCTITIS/
12. exp "INFLAMMATORY BOWEL DISEASES"/

13. (inflamm* ADJ2 (colon* OR bowel)).ti,ab
14. (ulcer* ADJ2 colitis).ti,ab
15. (pancolitis OR rectitis OR proctocolitis OR procto-colitis OR colorectitis OR rectocolitis OR rectocolitis OR recto-sigmoiditis OR rectosigmoiditis OR procto-sigmoiditis OR proctosigmoiditis OR proctitis).ti,ab
16. ((total OR sub-total OR subtotal OR extensive OR left-sided OR universal) ADJ colitis).ti,ab
17. (10 OR 11 OR 12 OR 13 OR 14 OR 15 OR 16)
18. ((intestin* OR gastrointestin* OR colon* OR bowel*) ADJ2 (motility OR sensitiv* OR functional OR irritable OR irritat* OR gas* OR spastic* OR unstable OR instability OR spasm*)).ti,ab
19. (flatus).ti,ab
20. (bloat*).ti,ab
21. (irritable bowel syndrome).ti,ab
22. (IBS).ti,ab
23. ((faecal OR fecal) ADJ2 incontinen*).ti,ab
24. (dyspepsia).ti,ab
25. exp "GASTROINTESTINAL MOTILITY"/
26. exp FLATULENCE/
27. exp "IRRITABLE BOWEL SYNDROME"/
28. exp "COLONIC DISEASES, FUNCTIONAL"/
29. exp "FECAL INCONTINENCE"/
30. (18 OR 19 OR 20 OR 21 OR 22 OR 23 OR 24 OR 25 OR 26 OR 27 OR 28 OR 29) 112092
31. (diarrhoea OR diarrhea).ti,ab
32. exp DIARRHEA/
33. (constipat*).ti,ab
34. exp CONSTIPATION/
35. (31 OR 32 OR 33 OR 34)
36. (30 OR 35)
37. exp CONSTIPATION/
38. (constipat*).ti,ab
39. exp "FECAL IMPACTION"/
40. ((feces OR faeces OR fecal OR faecal) ADJ3 (impaction OR impacted)).ti,ab
41. (difficult* OR delay* OR irregular* OR infrequen* OR pain*).ti,ab
42. (defecat* OR stool* OR faeces OR feces OR bowel movement*).ti,ab
43. (41 ADJ3 42).ti,ab
44. (f?ecalith? OR coprolith? OR stercolith?).ti,ab
45. (coprostasis).ti,ab
46. (obstipation).ti,ab
47. exp "FECAL INCONTINENCE"/
48. exp ENCOPRESIS/
49. (f?ecal incontinence OR soiling OR encopresis).ti,ab
50. exp MEGACOLON/
51. (megacolon OR megarectum).ti,ab
52. (37 OR 38 OR 39 OR 40 OR 43 OR 44 OR 45 OR 46 OR 47 OR 48 OR 49 OR 50 OR 51)
53. exp POLYPHENOLS/

54. (polyphenol* OR phenolic* OR flavon* OR flavan* OR catechin* OR isoflavon* OR anthocyanin* OR proanthocyanidin* OR procyanidin* OR ellagitannin* OR tannin* OR theaflavin* OR thearubigin OR "Phenolic acid*" OR "hydroxycinnamic acid*" OR "dihydroxycinnamic acid" OR trimethoxyphenol OR ferulic OR "hydroxybenzoic acid*" OR lignan* OR phytoestrogen OR secoisolariciresinol OR matairesinol OR stilben* OR resveratrol OR curcumin OR xanthohumol OR hydroxytyrosol OR quercetin OR rutin OR hesperidin OR hesperetin OR naringin OR narirutin OR naringenin OR daidzein OR genistein OR genistin OR daidzin OR epicatechin OR catechin OR epigallocatechin OR "epigallocatechin gallate" OR EGCG OR "chlorogenic acid*" OR "caffeic acid*" OR "coumaric acid*" OR phloretin OR cyanidin OR malvidin OR phenylvalerolactone* OR valerolactone* OR "phenylvaleric acid*" OR "valeric acid*" OR urolithin* OR equol OR enterolactone OR enterodiol OR enterolignan* OR "phenylpropionic acid*" OR "phenylpropanoic acid*" OR "phenylacetic acid*" OR "protocatechuic acid*" OR dihydrocaffeic OR dihydroferulic OR "hippuric acid*" OR pyrogallol OR hydroxytyrosol OR "homovanillic acid*" OR "protocatechuic acid*" OR protocatechualdehyde OR vanillin OR "vanillic acid*" OR 2-5-dihydro-benzoic-acid OR "dihydroxyphenyl propanoic acid*" OR "ellagic acid*").ti,ab
55. (poly ADJ2 phenol*).ti,ab
56. exp FLAVONOIDS/
57. exp LIGNANS/
58. exp STILBENES/
59. exp ANTHOCYANINS/
60. (tea OR "pu-er tea" OR "pu-erh tea" OR "Oolong tea" OR "black tea" OR "green tea" OR "red wine" OR grape OR grapeseed OR blueberr* OR cranberr* OR strawberr* OR blackberr* OR raspberr* OR blackcurrant OR berry OR cocoa OR chocolate OR coffee OR apple OR cider OR beer OR orange OR citrus OR grapefruit OR mango OR soy* OR "olive oil" OR flaxseed OR walnut* OR nuts OR almond OR pomegranate OR plum OR cherry OR potato OR sweet potato OR beans OR lettuce OR cabbage OR broccolis OR onion OR botanicals OR rosemary OR sage OR oregano OR clove OR cinnamon OR curry* OR coriander OR parsley OR thyme OR ginger).ti,ab
61. (53 OR 54 OR 55 OR 56 OR 57 OR 58 OR 59 OR 60)
62. (9 AND 61)
63. (17 AND 61)
64. (36 AND 61)
65. 62 [Human age groups Young adult OR Adult OR Middle Aged OR Aged OR Aged,80 and over] [Languages English] [Humans]
66. 63 [Human age groups Young adult OR Adult OR Middle Aged OR Aged OR Aged,80 and over] [Languages English] [Humans]
67. 64 [Human age groups Young adult OR Adult OR Middle Aged OR Aged OR Aged,80 and over] [Languages English] [Humans]

Search strategy: Cochrane
01. "polyphenol" AND "irritable bowel syndrome"
02. "polyphenol" AND "inflammatory bowel disease"
03. "polyphenol" AND "ulcerative colitis"
04. "polyphenol" AND "Crohn′s disease″
